# Natural weak value amplification in Fano resonance and giant Faraday rotation in magneto-plasmonic crystal

**DOI:** 10.1038/s41598-020-68126-8

**Published:** 2020-07-10

**Authors:** Shyamal Guchhait, Athira B S, Niladri Modak, Jeeban Kumar Nayak, Anwesha Panda, Mandira Pal, Nirmalya Ghosh

**Affiliations:** 1 Department of Physical Sciences, Indian Institute of Science Education and Research (IISER) Kolkata, Mohanpur, 741246 India; 2 Center of Excellence in Space Sciences India, Indian Institute of Science Education and Research (IISER) Kolkata, Mohanpur, 741246 India

**Keywords:** Optics and photonics, Physics

## Abstract

The extraordinary concept of weak value amplification can be formulated within the realm of wave interference as nearly destructive interference between the eigenstates of the measuring observable. Here we report on a phenomenon of interferometric weak value amplification of small polarization rotation in Fano resonance that evolves completely *naturally* due to near destructive spectral domain interference between a continuum and a narrow resonance mode having slightly different polarization response. In order to elucidate this, we first experimentally demonstrate an interferometric weak value amplification concept by generating nearly destructive interference of two paths of an interferometer having slightly rotated linear polarization states of light. The weak value amplification of polarization rotation effect is manifested as dramatic changes in the polarization state of light, which acts as the pointer. We go on to demonstrate that the manifestation of natural interferometric weak value amplification is an important contributing factor to the observed giant Faraday rotation and ellipticity in waveguided magneto-plasmonic crystals exhibiting prominent Fano resonance. The natural weak value interpretation of the enhanced Faraday rotation in hybrid magneto-plasmonic systems enriches the existing understanding on its origin. This opens up a new paradigm of natural weak measurement for gaining fundamental insights and ensuing practical applications on various weak interaction effects in rich variety of wave phenomena that originate from fine interference effects.

## Introduction

The weak measurement concept, introduced by Aharonov, Albert, and Vaidman^1–5^ involves three steps, quantum state preparation (pre-selection), a weak coupling between the pointer (device) and the measuring observable, and post-selection on a final state which is *nearly* orthogonal to the initial state^1–3^. The outcome, the so-called weak value may lie far outside the eigenvalue spectrum of an observable and can also assume complex values. These strange characteristics have allowed a wide range of applicability of weak values in both classical and quantum contexts^6–15^. The weak value amplification (WVA) has turned out to be a useful tool for addressing foundational questions in quantum mechanics^12,13^ and for resolving quantum paradoxes^14,15^. WVA is also finding widespread metrological applications^6–11^, to quantify small physical parameters, e.g., for precision measurements of angular rotation^6^, phase shift^8^, temporal shift^9^, frequency shift^10^, detection of ultra-sensitive beam deflections^11^, and so forth.

Even though WVA is a quantum mechanical concept, it can be understood using the wave interference phenomena and can therefore be realized in classical optical setting also^2,3,6–11^. In most of the optical weak measurements, Gaussian spatial modes of laser beams or Gaussian temporal pulse are employed as external pointer and associated polarization state of light is used as a pre-post selection mechanism, with tiny polarization dependent optical effects providing the weak coupling between the pointer and the measuring observable^3,6,7,9,10^. The enigmatic concept of WVA can also be interpreted as near destructive interference between the eigenstates of the measuring observable as a consequence of nearly mutually orthogonal pre and post selections of the system states^2,3^. Here, we demonstrate that due to the common interferometric origin, weak value amplification of an appropriate weak interaction parameter can naturally evolve in a universal wave interference phenomenon, namely, Fano resonance^16–20^. Fano resonance exhibits a characteristic asymmetric spectral line shape which emerges due to the interference of a discrete excited state with continuum of states^16^. Investigations on Fano resonances in wide range of micro and nano scale optical systems have attracted particular attention due to their numerous potential application such as in sensing, switching, lasing, filters, color display, nonlinear and slow-light devices and in invisibility cloaking etc^16,21–27^. The waveguided plasmonic crystal is one such nano-optical system that exhibit prominent Fano resonances and has already shown a number of interesting fundamental optical effects and demonstrated promising applications^16–18,20,26,27^. Taking example of Fano resonance in such a waveguided magneto-plasmonic crystal system^28–31^, we show that spectral domain interference between a continuum mode and a narrow resonance mode having slightly different polarization response can indeed provide a *natural* interferometric WVA of *weak* polarization rotation effect. In order to prove this concept, we first develop and experimentally demonstrate an intuitive interferometric WVA concept that uses near destructive interference of two paths of an interferometer having slightly rotated linear polarization states of light (playing the role of weak interaction). Unlike conventional optical weak measurements, here, near destructive interference between two paths are used to mimic the near orthogonal pre and post-selections of states (small overlap of states $\epsilon$) and polarization state of light is used as pointer. Real and imaginary WVAs of extremely small polarization rotation effect is obtained using small amplitude and phase offsets ($\epsilon_{a/p}$) of the waves in near destructive interference, respectively. The corresponding WVAs are manifested as $\epsilon$-dependent dramatic changes in the polarization state pointer profile, leading to large rotation of the polarization vector orientation angle and large changes in the circular (elliptical) polarization descriptor Stokes vector element of light^32^, respectively. We then show that a similar situation of WVA of small Faraday rotation naturally arises near the Fano spectral dip corresponding to the destructive Fano interference between an optically active (having small Faraday rotation) narrow quasiguided mode and a polarization isotropic photon continuum or broad surface plasmon mode in waveguided magneto-plasmonic crystal^28^. Using a theoretical model of interferometric weak value amplification and by employing finite element method (FEM) simulation^33^ in magneto-plasmonic crystal, we demonstrate that natural interferometric WVA plays an important role in the observed giant Faraday rotation in such systems that exhibit prominent Fano resonance features^28^. This is the first demonstration of weak value amplification that occurs completely naturally.

## Results and discussions

### Interferometric weak value amplification using polarization state as a pointer

We consider near destructive interference of two paths of an interferometer having slightly rotated (by a small angle $\alpha$) linear polarizations of light. The corresponding interfering electric field vectors for the two paths are

1 $${\varvec{E}}_{1} = E_{0} \hat{\user2{x}};\;\;{\varvec{E}}_{2} = E_{0} (\cos \alpha \hat{\user2{x}} + \sin \alpha \hat{\user2{y}})$$

The real and the imaginary WVA of the polarization rotation effect can be obtained by nearly destructive interference of ${\varvec{E}}_{1}$ and ${\varvec{E}}_{2}$ with small amplitude offset $\left( { \pm 2\epsilon_{a} } \right)$ and small phase offset $\left( { \pm 2\epsilon_{p} } \right)$, respectively^2, 3^. Here, $\epsilon_{a}$ is related to the amplitude ratio *a* of the two paths $\left( {\epsilon_{a} \approx \frac{1 - a}{{1 + a}}} \right)$. The corresponding expressions for the resultant electric field for the real and the imaginary WVAs are

2 $$\left[ {\left( {1 \pm \epsilon_{a} } \right){\varvec{E}}_{1} - \left( {1 \mp \epsilon_{a} } \right){\varvec{E}}_{2} } \right];\;\;\;\left[ {e^{{ \pm i\epsilon_{p} }} {\varvec{E}}_{1} - e^{{ \mp i\epsilon_{p} }} {\varvec{E}}_{2} } \right]$$

The expressions for the $\epsilon$-dependent variations of the resultant electric fields can be worked out using Eq. (1) into Eq. (2). It is convenient to represent the corresponding changes in the polarization state of light (acting as the pointer here) using the intensity-based Stokes vectorelements^32^.Using the weak interaction limit $\left( {\alpha \to 0} \right)$ it can be shown that the real and the imaginary WVAs of small polarization rotation effect $\alpha$ are manifested as $\epsilon$-dependent dramatic changes in the orientation angle ($\psi$) of the polarization vector and the circular (elliptical) polarization descriptor 4th Stokes vector element $\left( {\frac{{V}}{{I}}} \right)$ of light as

3a $$\psi = \frac{1}{2}{\tan}^{ - 1} \left( \frac{U}{Q} \right) \approx \pm \alpha {\cot}\epsilon_{a} \sim \pm \frac{\alpha }{{\epsilon_{a} }}$$

3b $$\frac{V}{I} \approx \pm \alpha cot\epsilon_{p} \sim \pm \frac{\alpha }{{\epsilon_{p} }}$$

As evident, as $\epsilon$ becomes small, the polarization vector orientation $\psi$ and the $\frac{ V}{I}$ Stokes parameter increase rapidly ($\propto \alpha cot\epsilon \sim \frac{\alpha }{\epsilon }$ in the limit of small $\epsilon$). Interestingly, like in the conventional linear response regime of WVA^2, 4^, here also there is a limit on the minimum value of $\epsilon \left( {\epsilon_{min} \sim 2\alpha } \right)$ where Eq. (3) is valid, which accordingly sets a bound on the maximum achievable amplification.

**Figure 1:**
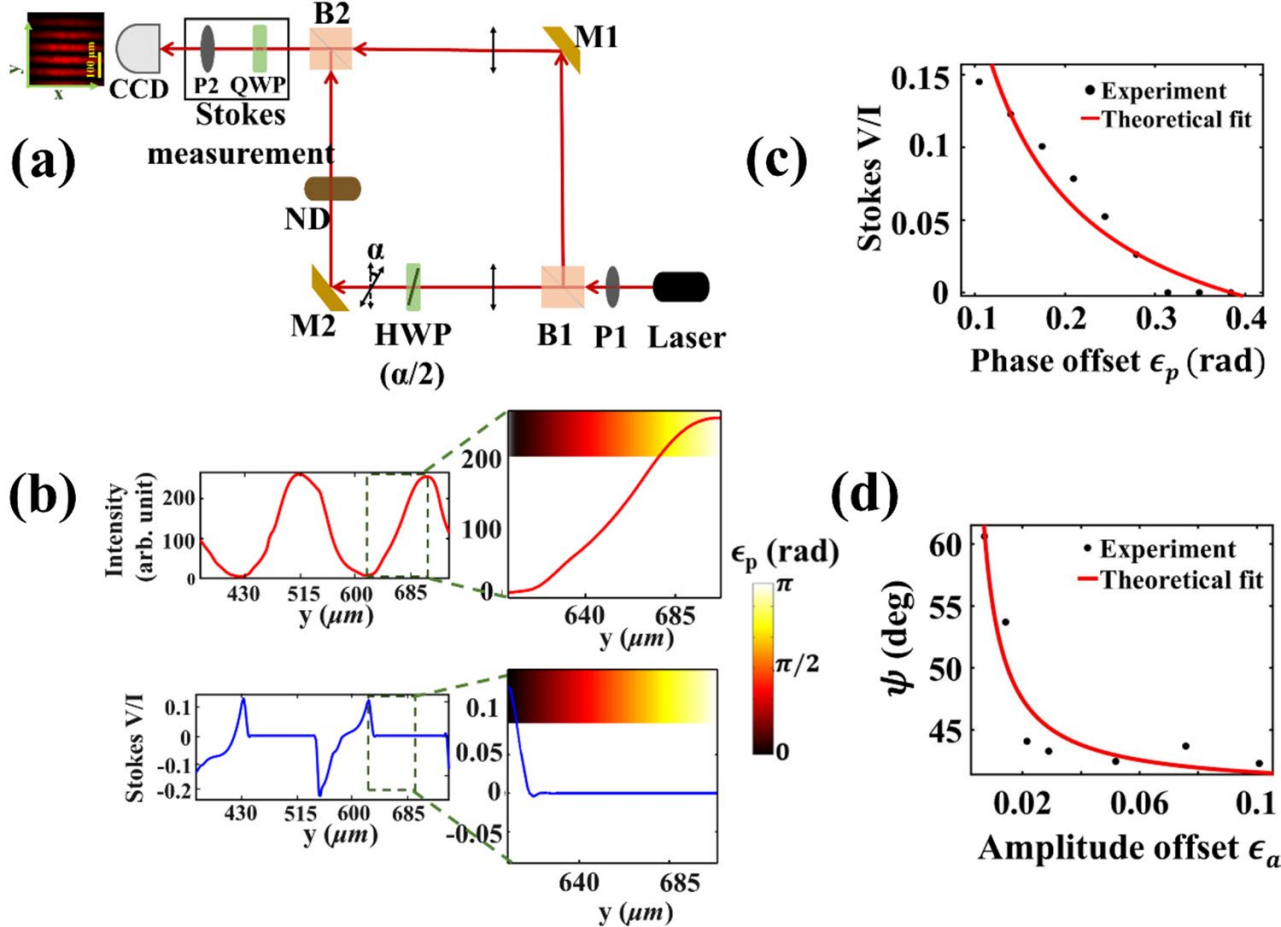
Interferometric weak value amplification using polarization state as a pointer. (**a**) A schematic of the Mach–Zehnder interferometric arrangement. A half waveplate (HWP) on one of the arms introduces a small polarization rotation $\alpha$ as the weak interaction effect. P1, P2: linear polarizers, B1, B2: beam splitters, M1, M2: mirrors, ND: Variable neutral density filter. QWP: Quarter waveplate. (**b**) and (**c**) The results of imaginary weak value amplification. (**b**) The spatial (along the *y*-direction shown in the inset of a) variation of the intensity (top panel) and the corresponding $\frac{V}{I}$ Stokes vector element (bottom panel). The magnified views of the variations around the position of the intensity minima (destructive interference with phase difference $\pi$) are shown along with the phase offset from $\pi , \epsilon_{p}$ (shown by the color bar). (**c**) The corresponding variation of $\frac{V}{I}$ with $\epsilon_{p}$ (closed circle) and theoretical fit to Eq. (3b) (red line, fitted value of $\alpha = 1.16$°). (**d**) The results of real weak value amplification. The variation of the polarization vector orientation angle $\psi$ (closed circle) as a function of the amplitude offset parameter $\epsilon_{a}$ recorded at the spatial position of intensity minima corresponding to the destructive interference. The theoretical fit to Eq. (3a) is shown by red line (fitted $\alpha = 1.13$°).

We first experimentally demonstrate this WVA concept using a conventional Mach–Zehnder interferometric arrangement (Fig. 1a, see “Methods”). The corresponding results are summarized in Fig. 1. The $\frac{V}{I}$ Stokes polarization parameter is observed to exhibit clear increasing trend as one approaches the position of the intensity minima in the fringe pattern (Fig. 1b). The variation of $\frac{V}{I}$ with the small phase offset parameter $\epsilon_{p}$ (Fig. 1c) accordingly showsgood agreement with the corresponding prediction of Eq. (3b) $\left( { \propto \alpha cot\epsilon_{p} \sim \frac{\alpha }{{\epsilon_{p} }}} \right)$, demonstrating faithful imaginary WVA. The polarization vector orientation angle ($\psi$), determined from the measured $U\,{\text{and}} \ Q$ Stokes parameters also scales as $\left( { \propto \alpha cot\epsilon_{a} \sim \frac{\alpha }{{\epsilon_{a} }}} \right)$ with decreasing amplitude offset parameter $\epsilon_{a}$ (Fig. 1d), confirming real WVAof polarization rotation effect $\alpha$ (Eq. 3a). These and other experimental results for varying $\alpha$ validated this new interferometric concept of WVA that uses polarization state as a pointer and near destructive path interference as pre-post selection mechanism.

### Natural weak value amplification using spectral interference of Fano resonance

We now proceed to show that the above interferometric WVA concept *naturally* arises in the spectral domain interference of optical Fano resonance. For this purpose, we model the Fano resonance as a coherent interference of a narrow resonance mode described by a complex Lorentzian $\left( {L^{R} \left( \omega \right)} \right)$ with a frequency-independent ideal continuum^17–19^. Moreover, we assume that the polarization of the narrow resonance mode is rotated by a small angle $\alpha$ with respect to the *y*-polarized continuum mode and also has a small ellipticity $\chi$. The corresponding polarized filed can be expressed as

4 $$\begin{aligned} {\varvec{E}}_{{\varvec{s}}} \left( {\upomega } \right) & \approx \left[ {L^{R} \left( \omega \right)\left( {{{ \cos\upalpha }}\cos \chi - i\sin \alpha \sin \chi } \right)\hat{\user2{y}} + \left( {\sin \alpha \cos \chi + i\cos \alpha \sin \chi } \right)\hat{\user2{x}} + \hat{\user2{y}}} \right] \\ & = \left[ {\frac{q - i}{{\epsilon \left( \omega \right) + i}}\left( {{{ \cos\upalpha }}\cos \chi - i\sin \alpha \sin \chi } \right)\hat{\user2{y}} + \left( {\sin \alpha \cos \chi + i\cos \alpha \sin \chi } \right)\hat{\user2{x}} + \hat{\user2{y}}} \right] \\ \end{aligned}$$

Here, ${{ \upvarepsilon }}\left( {\upomega } \right) = \frac{{{\upomega } - {\upomega }_{0} }}{{\left( {{\upgamma }/2} \right)}}$, ${{ \upomega }}_{0}$ and ${\upgamma }$ are the central frequency and the width of the narrow resonance and $q$ is the Fano asymmetry parameter related to the coupling of the modes^34–36^. Note that at the Fano frequency $\omega_{F} = \left( {\omega_{0} - \frac{q\gamma }{2}} \right)$ (corresponding to ${\upvarepsilon } = - q$), exact destructive interference takes place, where the phase difference $\left[ {{\Psi }\left( {\omega_{F} } \right)} \right]$ between the narrow resonance and the continuum mode becomes $\pi$ and their amplitude ratio approaches unity $\left( {a = 1} \right)$ (see Supporting information S2 for details of the model). As one moves *spectrally* slightly away from $\omega_{F} \left( {\omega = \omega_{F} \pm \delta } \right)$, the desirable near destructive interference scenario is obtained (like in Eq. 2) but now with simultaneous amplitude $\left( {\epsilon_{a} } \right)$ and phase $\left( {\epsilon_{p} } \right)$ offsets. The expressions for these offset parameters can be written as

5 $$\epsilon_{a} \left( \omega \right) \approx \frac{1 - a}{{1 + a}} = \frac{{\left( {1 - \sqrt {\left( {\frac{{q^{2} + 1}}{{{\upvarepsilon }^{2} + 1}}} \right)} } \right)}}{{\left( {1 + \sqrt {\left( {\frac{{q^{2} + 1}}{{{\upvarepsilon }^{2} + 1}}} \right)} } \right)}};\;\;\epsilon_{p} \left( \omega \right) = \pi - tan^{ - 1} \left[ {\frac{{q + {\upvarepsilon }}}{{1 - q{\upvarepsilon }}}} \right]$$

**Figure 2:**
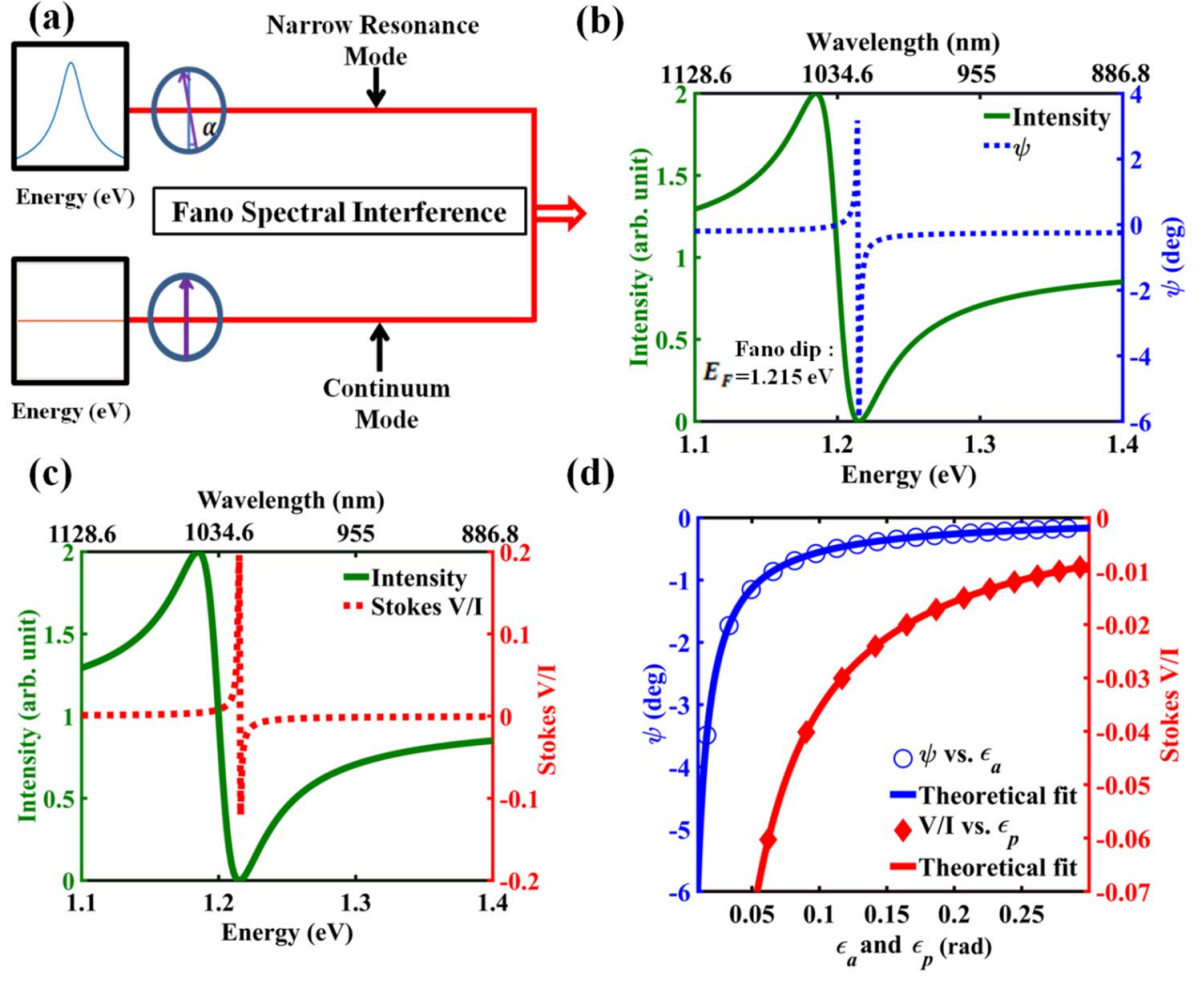
Schematic illustration of natural weak value amplification in the spectral domain interference of Fano resonance. (**a**) Interference of a narrow resonance mode with a continuum mode yields an asymmetric Fano spectral line shape of resonance. The small polarization rotation $\alpha$ provides the desirable weak coupling. (**b**) The theoretically calculated (using Eq. 4) spectral variation ($E = \hbar \omega = 1.1 - 1.4$ eV, corresponding to $\lambda =\,1,128.6-886.8\, nm$) of the Fano intensity profile (left axis, green solid line) and the polarization vector rotation angle $\psi$ (right axis, blue dashed line). The black arrow denotes the energy (frequency) corresponding to the exact destructive interference or the Fano spectral dip ($E_{F} = \hbar \omega_{F}$). (**c**) The variation of the $\frac{V}{I}$ Stokes vector element (right axis, red dashed line). Both the $\psi$ and the $\frac{V}{I}$ polarization parameters exhibit large enhancement as one approaches $E_{F}$. (**d**) The corresponding variations of the $\psi$ (left axis, blue open circle) and $\frac{V }{{I }}$ (right axis, red solid diamond) polarization parameters with the amplitude and the phase offset parameters $\epsilon_{a}$ and $\epsilon_{p}$ respectively. The theoretical fits to Eqs. E4a and E4b of the supporting information are shown by solid lines of the same colors. The following parameters of Fano resonance were used for these theoretical calculations: $E_{0} = 1.2 \;{\text{eV}}$, $\gamma = 0.03\;{\text{eV}}$, $q = - 1, \;E_{F} = 1.215 \;{\text{eV}}$ and $\alpha = 0.23$°, $\chi = 0$°.

The electric field of Fano resonance in Eq. (4) can be posed in the same form of the electric field of interferometric WVA of Eq. (2) using the small amplitude and phase offset parameters $\epsilon_{a/p}$ of Eq. (5) (see Eq. E2 of Supporting information S2), which sets the foundation for natural WVA in spectral interference of Fano resonance. Using the electric fields of Eq. (4), the expressions for the relevant Stokes vector elements can be derived and the dependence of the resulting $\psi$ and $\frac{V}{I}$ polarization parameters on $\epsilon_{a}$ and $\epsilon_{p}$ can be studied (see Eqs. E3 and E4 of Supporting information S2). In Fig. 2, we have schematically illustrated the spectral analogue of the interferometric WVA concept in Fano resonance (Fig. 2a) and presented the corresponding theoretical results using Eqs. (4) and (5) (Fig. 2b–d). As evident, both the polarization vector orientation angle ($\psi$) (Fig. 2b) and the $\frac{V}{I}$ Stokes polarization parameter (Fig. 2c) exhibit dramatic enhancement at close vicinity of the Fano spectral dip (Energy $E_{F} = \omega_{F}$) corresponding to near destructive spectral domain interference. As one moves *spectrally* away from $E_{F}$, the $\epsilon_{a}$ and $\epsilon_{p}$ parameters change simultaneously and consequently the $\psi$ and the $\frac{V}{I}$ polarization parameters scale as $\left( {\sim \alpha cot\epsilon_{a/p} \sim \frac{\alpha }{{\epsilon_{a/p} }}} \right)$ simultaneously with both the offset parameters $\epsilon_{a/p}$ (Fig. 2d), as a manifestation of natural weak value amplification. It might be worth noting here that in our previous study, in non-magnetic waveguided plasmonic crystal systems^18^, we had demonstrated an interesting WVA scheme using the asymmetric spectral line shape of Fano resonance as a pointer which was different from conventional pointers in optical WVA schemes such as the Gaussian spatial modes of laser beam^3,6,7,9,10^. Pre and post selection using near orthogonal linear and elliptical polarization states in that WVA scheme yielded spectacular tunability of Fano resonance line shape^18^. In contrast, in the current scheme, WVA of a small polarization anisotropy effect *naturally* arises due to near destructive spectral domain Fano interference of a continuum mode and a narrow resonance mode having slightly different polarization response, as evident from Eq. (4), (5) and Eqs. (E2–E4) of Supporting information.

**Figure 3:**
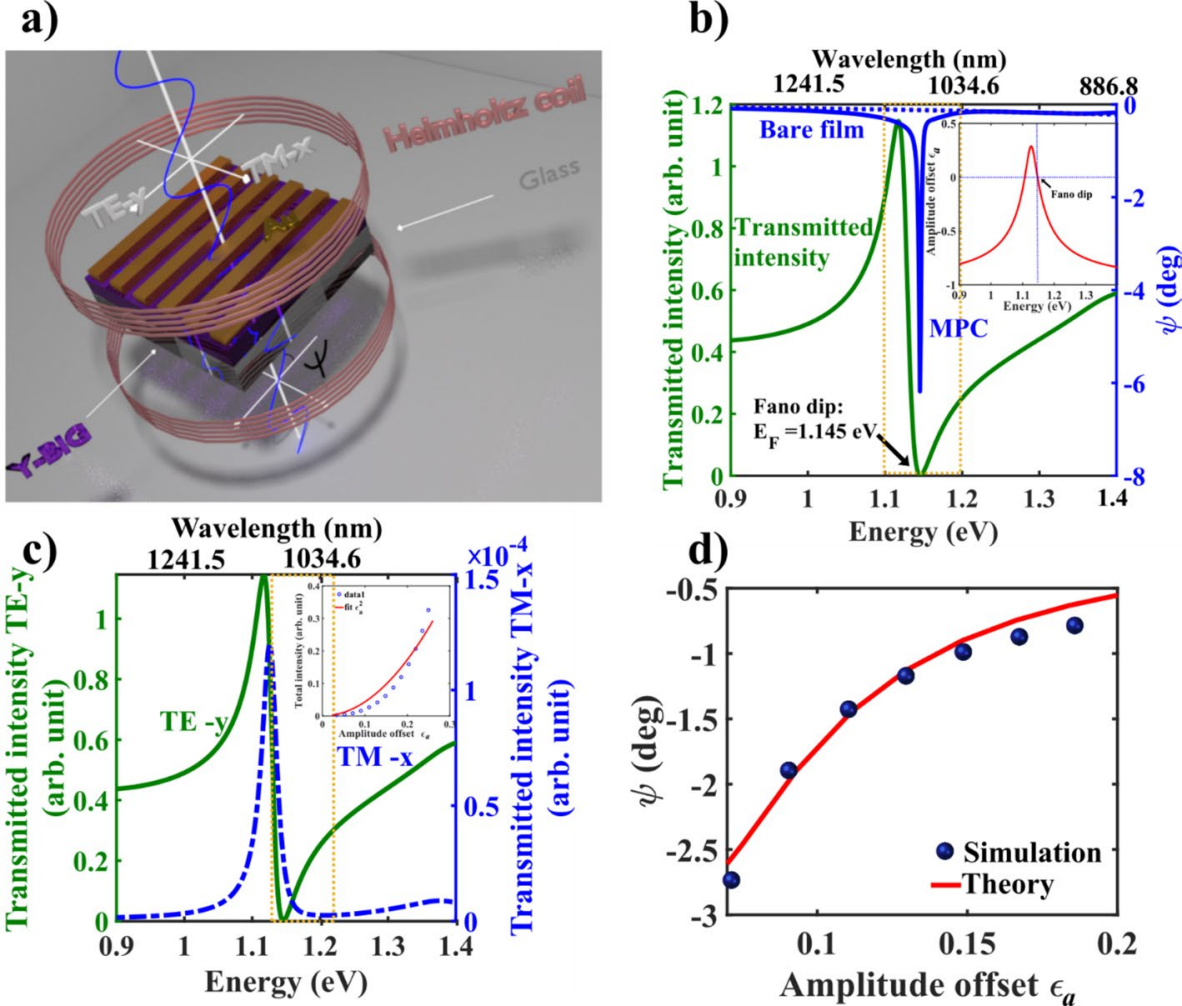
Natural weak value amplification of Faraday rotation in Fano resonant waveguided magneto-plasmonic crystal. (**a**) Schematic illustration of FEM simulation of Fano resonance and Faraday rotation in magneto-plasmonic crystal. (**b**) The transmitted intensity (left axis, green line) exhibits prominent Fano spectral asymmetry ($E = \hbar \omega = 0.9 - 1.4$ eV, corresponding to $\lambda =\,1,379.4-886.8\,nm$ shown here). The spectral variation of the polarization vector rotation angle $\psi$ (enhanced Faraday rotation) of the magneto-plasmonic crystal (right axis, blue solid line) is shown along with the corresponding rotation for a bare Y-BIG film (right axis, blue dashed line). The spectral variation of amplitude offset parameter $\epsilon_{a}$ is shown in the inset. (**c**) The spectral variation of the transmitted TE-$y$ (left axis, green solid line) and TM-$x$ polarized intensities (right axis, blue dashed line), with TE-$y$ polarization excitation. The Fano spectral dip region where the rotation reaches its maximum value is marked. The inset shows the variation of total intensity (open blue circle) with $\epsilon_{a}$ and a theoretical fit (red solid line) to $\sim \epsilon_{a}^{2}$ variation. (**d**)The natural weak value amplification of the polarization vector rotation angle $\psi$ (Faraday rotation) (blue solid balls) as a function of amplitude offset parameter $\epsilon_{a}$. The exact theoretical predictions (red solid line) of natural WVA (Eqs. E2 and E3 of Supporting information) in Fano resonance using a value for rotation of bare film $\alpha = 0.28$° and ellipticity $\chi = 0.25$°. The chosen morphological parameters of the magneto-plasmonic crystal (see text) resulted in the following parameters of Fano resonance: $E_{0} = 1.127\;{\text{ eV}},\; \gamma = 0.0267 \;{\text{eV}},\;q = - 1.084,\; E_{F} = 1.141 \;{\text{eV}}$.

We now demonstrate this concept in a realistic Fano resonant system, namely, the waveguided magneto-plasmonic crystals^28–32^. The waveguided plasmonic crystal system usually comprises of a periodic array of noble metal nanostructures (e.g., a metal grating in the system investigated here) on top of a dielectric waveguiding layer^16–18,20^. The coupling of the surface plasmons in the metallic nanostructures or the photon continuum with the narrow quasi-guided photonic modes in the waveguiding layer leads to Fano resonance^17,20,26–28^. It is pertinent to note that for excitation with transverse electric (TE) polarized light, the surface plasmons are not directly excited in such system as the electric field is parallel to the metal grating (subsequently shown in Fig. 3a, see “Methods”). In this case, the interference between the narrow quasi-guided photonic mode and the photon continuum (for excitation with a broad band light source) leads to the emergence of asymmetric spectral line shape of Fano resonance in the transmission profile^17,20,26,27^. For excitation with transverse magnetic (TM) polarization on the other hand, Fano type resonance appears due to the interference of the broad surface plasmon mode in the metal nanostructures and the quasi-guided photonic mode in the waveguiding layer^17,20,26,27^. As opposed to the usual plasmonic crystal systems, in waveguided magneto-plasmonic crystal, the waveguiding layer is made of magneto optical materials that additionally exhibit Faraday rotation and ellipticity^28–32^, which acts as the desirable weak interaction parameter in this weak measurement scenario of Fano resonance. We demonstrate below that the in-built near destructive spectral domain Fano interference in the neighborhood the Fano spectral dip in such systems provides the near orthogonal pre-post selection of states leading to *natural* interferometric WVA of small Faraday rotation and ellipticity. We also show that this *natural* interferometric WVA of Fano resonance is an important contributing factor to the recently observed giant enhancement of small Faraday rotation in such system^28^. We used finite element method (FEM)^33^ for simulating the Fano resonance in the transmitted light from magneto-plasmonic crystal system (Fig. 3a, see “Methods”) and the results are presented here for input TE(*y*) polarization excitation. Figure 3 summarizes the corresponding results of natural WVA of Faraday rotation in Fano resonance for TE polarized excitation. The transmission spectra exhibit prominent Fano resonance with characteristic asymmetric spectral line shape (Fig. 3b). The derived Fano resonance parameters, namely, the central frequency (or energy $E = \hbar \omega$) of narrow resonance $E_{0} \left( {\hbar \omega_{0} } \right),$ the width of the narrow resonance $\gamma$, and the asymmetry parameter $q$ are noted in the figure caption, which are found to be comparable to those previously reported for similar plasmonic crystals samples^17,18,20,27,28^. Before we proceed to the main findings of this study on natural interferometric WVA in Fano resonance, it is worth noting that the waveguide modes in the magneto-plasmonic crystal system are leaky as they couple to light through momentum matching enabled by the presence of the gold (Au) grating on top of the waveguide layer (Y-BIG film)^20^. The overall damping and the lossy nature of such quasiguided photonic modes are accordingly reflected in the comparatively large line width $\gamma$ of Fano resonance (Fig. 3b and caption), which is also a sensitive indicator of the typical de-phasing time of the resonant modes in such systems that is known to be inversely related with the $\gamma$ parameter^37^. Now, turning to the results on the natural interferometric WVA, significant enhancement of the Faraday rotation (polarization vector orientation angle $\psi$) is observed near the spectral window of the Fano dip corresponding to near destructive interference of the narrow quasiguided resonance mode and the broad photon continuum (Fig. 3b). This variation is subsequently interpreted in terms of the small amplitude offset parameter $\epsilon_{a}$ (its spectral variation is shown in the inset) in the weak value formalism of Fano interference (Eq. 5). Note that, for input TE polarization, the surface plasmons are not directly excited. In this case the known mechanism, for the enhancement of Faraday effects is the so-called resonance-enhanced cross-coupling between the TE ($y$) and the TM ($x$) quasiguided modes due to their strong spectral overlap^28–31,38^. However, Fig. 3c clearly shows that with TE ($y$) polarized excitation, the transmitted TM ($x$) polarized intensity is several orders weaker than the TE polarization signal and both approach their minima in the vicinity of the Fano spectral dip where the rotation reaches its maximum value. This is a clear indication that near destructive interference of the TE polarized quasiguided mode with the continuum mode at close vicinity of the Fano spectral dip, or in other words, *natural interferometric* WVA is the underlying reason for the observed enhancement of Faraday rotation. Moreover, the amplification takes place at the expense of the total intensity signal and the corresponding $\sim \epsilon_{a}^{2 }$ intensity variation in this spectral region (inset of Fig. 3c) is a universal signature of WVA1,2,4. The corresponding variation of FEM simulated Faraday rotation $\psi$ also follows the $\left( { \propto \alpha cot\epsilon_{a} \sim \frac{\alpha }{{\epsilon_{a} }}} \right)$ behavior (Fig. 3d), which is the hallmark of WVA. A quantitative comparison of the amplified Faraday rotation $\psi$ with the exact theoretical predictions of natural WVA in Fano resonance (using Eqs. E2 and E3 of Supporting information S2) shows remarkable agreement (Fig. 3d). Fitting of $\psi$ with the natural WVA equation (Eq. E4a of Supporting information) yields a value of $\alpha = 0.34$° as compared to $\alpha = 0.28$° of the bare film. The $\frac{V}{I}$ Stokes ellipticity parameter also exhibited *natural* WVA with varying phase offset $\epsilon_{p} \left( {\sim \alpha cot\epsilon_{p} } \right)$ and showed excellent agreement with the corresponding theoretical predictions (see Supporting information Fig. S2). These results therefore provide conclusive evidence that for TE polarization excitation, the giant enhancement of Faraday rotation and ellipticity around the spectral window of Fano resonance in magneto-plasmonic crystal^28^ is a manifestation of *natural* interferometric WVA. In contrast, for TM polarization excitation, the competing roles of the different mechanisms, namely, the natural interferometric WVA, the enhancement of electromagnetic near fields, resonance enhanced cross-coupling between TE and TM quasi-guided modes etc. are rather complex due to the direct excitation of the surface plasmons^28–32,38^. The relative contributions of these crucially depends upon a number of parameters including the geometrical parameters of the structures, coupling strength of the plasmon and the waveguide modes, the purity of asymmetric spectral line shape of Fano resonance, avoided crossing behavior etc.^28,31,38^ (results not shown here). This is being investigated in details.

In general, it is exclusively in the case of prominent Fano resonance that the near destructive spectral domain interference between a continuum and a narrow resonance mode having slightly different polarization response mimics the unique phenomenon of natural interferometric WVA, which cannot be produced by any arbitrary interference effects. Caution must therefore be exercised in interpreting enhancements of magneto-optical effects in many other plasmonic systems which do not exhibit prominent Fano type spectral interference^29,31^. In such scenario, the natural WVA phenomenon does not take place and the enhancement of magneto optical effects can be primarily attributed to the other known mechanisms^28–31,38^. We also emphasize that the presented framework of natural WVA in spectral interference of Fano resonance is a generic one, which is applicable to a broad range of Fano resonant systems (irrespective of their geometrical or structural complexities) in the presence of an appropriate weak interaction parameter (like the small Faraday rotation in case of the magneto-plasmonic crystal). As evident, the ratio of the amplitudes of the narrow resonance mode and the continuum mode $\left( {a\left( \omega \right) = \sqrt {\frac{{{q}^{2} + 1}}{{\upvarepsilon^{2} + 1}}} } \right)$ and the phase difference between them $\left( {\Psi \left( \omega \right) = tan^{ - 1} \left[ {\frac{{\left( {q + \upvarepsilon } \right)}}{1 - q\upvarepsilon }} \right]} \right)$ (see Supporting information S2)^17,18^ are only important in the weak value formalism. Since these two parameters are directly related to the coupling strength or the resulting Fano asymmetry parameter $q$, information on these parameters and on the resulting WVA can easily be quantified from the recorded Fano spectral line shape of any system.

## Conclusions

In summary, we have developed and experimentally validated a fundamentally interesting concept of interferometric WVA to amplify small polarization anisotropy effect. Unlike conventional optical WVA schemes, in this approach, near destructive interference between two paths of an interferometer is used to mimic the near orthogonal pre and post-selections of states, small polarization anisotropy effects act as the weak interaction, and polarization Stokes parameters are used as pointer. It is shown that such interferometric WVA naturally arises in Fano resonance due to the spectral domain interference between a continuum mode and a narrow resonance mode having slightly different polarization response which arises due to the presence of a *weak* polarization anisotropy effect in one of the interfering modes. The results of these studies provide new insights on the vital role of natural WVA in the giant enhancement of Faraday rotation and ellipticity in Fano resonant magneto-plasmonic systems. In general, these results are quite remarkable as the weak value amplification procedure usually demands careful state preparation (pre-selection), a weak coupling between the pointer and the measuring observable and post-selection on a final state. Whereas these results clearly show that the entire process of WVA may naturally evolve in the spectral domain Fano interference in the presence of a weak polarization anisotropy effect. It is envisaged that in presence of an appropriate weak interaction parameter such natural interferometric WVA can be realized in diverse other non-trivial phenomena also that originate due to fine interference effects^27,39–41^. This opens up a new regime for the study of natural weak measurements on fundamentally important weak interactions associated with a rich variety of interference related effects for their enhanced probing and for enhancing related metrological applications in the optical domain and beyond.

## Methods

### Experimental method for interferometric weak value amplification using polarization state as a pointer

We employed a conventional Mach–Zehnder interferometric arrangement to demonstrate the interferometric WVA concept (Fig. 1a) (see Supporting information S1 for details). The fundamental Gaussian mode of 632.8 nm line of a He–Ne laser is linearly polarized (horizontal, ${\varvec{x}}$-polarization) using a polarizer (P1), which then enters the interferometer. A small polarization rotation $\alpha$ (1°.) is introduced in one of the arms of the interferometer by orienting the fast axis of a half waveplate (HWP, mounted on a high precision rotational mount) at an angle α/2. The interfering light beams after exiting the interferometer are passed through a combination of a quarter waveplate (QWP) and a linear polarizer (P2) and the polarization-resolved interference fringes are imaged into a CCD camera (2048 × 1536 square pixels, pixel dimension 3.45 μm). The quarter waveplate and the linear polarizer combination are used to determine the spatial variation of the four Stokes polarization parameters of light^32^ across the interference fringe. In order to realize the real WVA scheme, the relative intensities (or amplitude ratio *a*) of light in the two interfering paths were varied using a variable neutral density filter ND. The polarization vector orientation angle ($\psi$) at a spatial position corresponding to the intensity minima of the interference fringe was determined from the recorded spatial variation of the $U \;{\text{and}}\; Q$ Stokes parameters. The corresponding real WVA was probed by observing changes in the $\psi$-parameter as a function of varying amplitude offset parameter $\epsilon_{a}$ (or *a*). For probing the imaginary WVA, on the other hand, the intensities of the two paths were kept equal and the recorded $\frac{V}{I}$ Stokes parameter for varying spatial positions away from the point of the intensity minima or destructive interference was used (which demarcates the position for phase difference $\pi$). The measured spatial variation of $\frac{V}{I}$ was subsequently used to generate its variation as a function of the small phase offset (from $\pi$) parameter $\epsilon_{p}$.

### Finite element method (FEM) simulation of Fano resonance in waveguided magneto-plasmonic crystals

We used finite element method (FEM)^33^ for simulating the Fano resonance in the transmitted light from such system (Fig. 3a). Briefly, it consists of 1-D periodic grating of gold (Au) nanostructures on top of a Y-BIG film, acting as the waveguiding layer^28^. The dimension of the Au grating was (width $= 120\,nm$, centre to centre distance $= 600\,nm$ and height $= 65\,nm$) and the thickness of the Y-BIG film was 150 nm. The far field transmission spectra ($\lambda =\,887-1,380\,nm$) and its polarization dependence were studied (see Supporting information S3 for the details of the procedure). For the simulations, the permittivity tensor elements of the Y-BIG film were taken, $\epsilon_{11} = \epsilon_{33} = 6.7 + 0.053i$ and $g = 0.016 - 0.0092i$ for typical magnetic field of 140mT^28^ and dielectric permittivity of Au was taken from literature^42^. Anisotropy of the Y-BIG film arises due to the evolution of the off-diagonal elements in the corresponding permittivity tensor^28^.

## Supplementary information

Supplementary information

## Data Availability

The datasets generated during and/or analyzed during the current study are available from the corresponding author on reasonable request.
