# Supplementary material for: Natural weak value amplification in Fano resonance and giant Faraday rotation in magneto-plasmonic crystal
Source: Sci Rep. 2020 Jul 10;10:11464. doi: 10.1038/s41598-020-68126-8 (PMC7351789; doi:10.1038/s41598-020-68126-8)
Supplement: Supplementary file 1 — Supplementary information [file 41598_2020_68126_MOESM1_ESM.docx]

**Supporting Information**

**Natural weak value amplification in Fano resonance and giant Faraday rotation in magneto-plasmonic crystal**

Shyamal Guchhait^1^, Athira B S^2^, Niladri Modak^1^, Jeeban K Nayak^1^, Anwesha Panda^1^, Mandira Pal^1^, Nirmalya Ghosh^1,2*^

*^1^Department of Physical Sciences,Indian Institute of Science Education and Research (IISER) Kolkata, Mohanpur 741246, India*

*^2^Center of Excellence in Space Sciences India,Indian Institute of Science Education and Research (IISER) Kolkata, Mohanpur 741246, India*

**Corresponding authors:nghosh@iiserkol.ac.in*

***S1. Experimental details of Interferometric weak value amplification***

In conventional optical weak measurement schemes, the polarization state of light is used for pre and post selection of states and small polarization dependent effects such as beam deflection, phase shift or spectral shift are used as weak interaction effect [1, 2]. In contrast to these traditional schemes, we have described an interferometric weak value amplification (WVA) scheme that uses path interference as pre-post selection mechanism, small polarization rotation (or anisotropy) effect as weak interaction and the intensity-based Stokes polarization parameter as the pointer. The fundamental Gaussian (TEM_00_) mode of 632.8 nm line of a He–Ne laser (HRR120-1, Thorlabs, USA), was spatially filtered and collimated using Lens, pinhole and aperture assembly wasthen used to seed the interferometric systemshown in Figure 1(a) of the main text. The spatial variation of the polarization state of the light at the exit end of the interferometer was analyzed by experimentally recording the four Stokes polarization parameters [*I, Q, U, V*] [3] at the CCD image plane. Standard measurement procedure [3] was adopted for this purpose by performing six linear and circular polarization intensity measurements usinga combination of linear polarizer and quarter wave plate:*I_H_*– horizontal linear polarization (0^°^); *I_V_* – vertical linear polarization (90°);*I_P_* –45° linear polarization; *I_M_* – 135° linear polarization;*I_R_ –* right circular polarizer (polarizer at 0° and quarter waveplate at 45°); *I_L_–* left circular polarizer (polarizer at 0° and quarter waveplate at 135°).

As described in the main text, for studying the imaginary WVA, a single set of measurement of the spatial variation of the Stokes *V/I* parameter was sufficient. For this measurement, the intensities of the two paths were kept equal (amplitude ratio *a =1*) and the spatial variation of the *V/I* Stokes polarization parameter around the destructive interference point (phase difference$\pi$) was studied as a function of the phase offset (from $\pi$) parameter$\epsilon_{p}$. Typical measurement of the spatial variation of the Stokes V/I across the minima and the maxima of the interference fringe is shown in Figure **S1**(corresponding to the results shown in Figure 1c). Probing the real WVA,on the other hand, involved multiple set of measurements of the spatial variation of Stokes polarization parameters *Q* and *U* for varying intensity ratio (varying $a$)of the two arms. For each value of $a$,the measured *Q* and *U* Stokes polarization parameters at a spatial position corresponding to the intensity minima (destructive interference point) was used to generate the dependence of the polarization rotation angle $\psi=\frac{1}{2}{tan}^{-1}\left( \frac{U}{Q} \right)$on the small amplitude offset parameter $\epsilon_{a}\left( \approx\frac{1-a}{1+a} \right)$as shown in Figure 1(d) of main text.

**
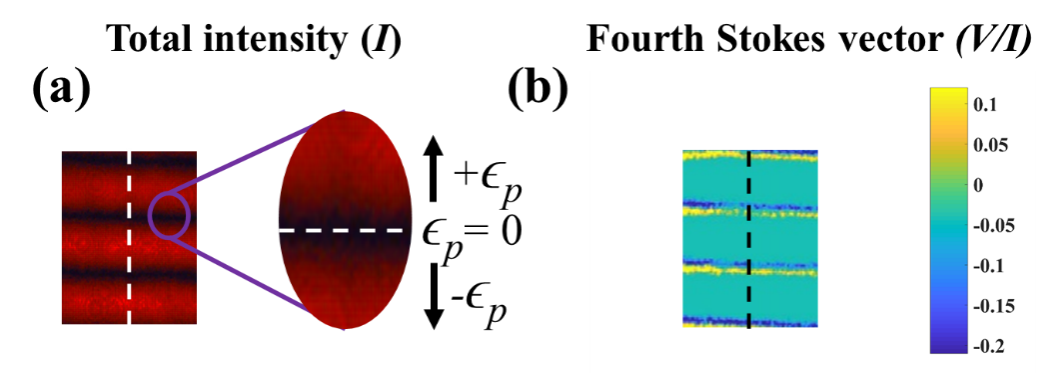
**

**Figure S1:**Experimentally measured images of the (a) total intensity*(I)*and (b)$\frac{V}{I}$ Stokes polarizationparameteracross the interference fringe. At the point of destructive interference the phase offset parameter $\epsilon_{p}$ becomes zero and increases if we go off from the destructive interference point.The spatial variation of $\frac{V}{I}$ at close vicinity of the intensity minima (or destructive interference) was used to study the imaginary weak value amplification through the dependence of $\frac{V}{I}$with the phase offset parameter $\epsilon_{p}$ (in Fig. 1b and 1c). The dotted line corresponds to the line profile of $\frac{V}{I}$ shown in Fig. 1b.

***S2.* Theoretical model of natural weak value amplification in Fano resonance**

Our natural weak value amplification (WVA) formalism is founded on a simple but intuitive model of optical Fano resonance (Eq. 4 of the main text) that uses coherent interference of a narrow resonance mode described by a complex Lorentzian with afrequency-independent continuum (or broad) mode [4,5]. It has been observed earlier that similar simple model can capture the near field mode coupling and the Fano interference effect in various optical systems including the plasmonic crystals [4-10]. Here the spectral domain interference betweenthe modes is described through relevant phase terms where the phase difference between two modes $\Psi(\omega)$ comprises of two factors, the Fano phase $\varphi_{F}$, (related to mode coupling and the resulting Fano spectral asymmetry $q$) and the phase associated with the narrow resonance$\theta\left( \omega\right).$ The phase terms are described as [4]

$$\varphi_{F}=-{tan}^{-1}\left( \frac{1}{q} \right)$$

$$\theta\left( \omega\right)=-{tan}^{-1}\left( \frac{1}{\epsilon} \right)$$

${\Psi\left( \omega\right)=tan}^{-1}\left[ \frac{\left( q+\epsilon\right)}{1-q\epsilon} \right]$ (E1)

Note that the ratio of the amplitudes of the narrow resonance mode and the continuum mode (in Eq. 4) approaches unity $\left( a\left( \omega\right)=\sqrt{\frac{q^{2}+1}{\epsilon^{2}+1}}=1 for \varepsilon=-q \right)$ and the phase difference $\Psi\left( \omega_{F} \right)=\pi$at the Fano frequency $\omega_{F}$ corresponding to the exact destructive interference.

The expression for the electric field of Fano resonance relevant to our natural WVA formalism was provided in Eq. 4 of the main text. In the neighbourhood of the Fano destructive interference $\left( \omega=\omega_{F}\pm\delta\right)$,the expression for the electric field described in Eq. 4 of the main text can be written in terms of the small amplitude $\left( \epsilon_{a} \right)$and phase $\left( \epsilon_{p} \right)$offset parameters (defined in Eq. 5 of the main text) as

$\boldsymbol{E}_{\boldsymbol{s}}\left( \omega\right)\boldsymbol{=}\left[ \left( 1+\epsilon_{a} \right)e^{+i\epsilon_{p}}\left\{ \left( \cos\alpha\cos\chi-i\sin\alpha\sin\chi\right)\hat{\boldsymbol{y}}\boldsymbol{+}\left( \sin\alpha\cos\chi+i\cos\alpha\sin\chi\right)\hat{\boldsymbol{x}} \right\}\boldsymbol{-}\left( 1-\epsilon_{a} \right)e^{-i\epsilon_{p}}\hat{\boldsymbol{y}} \right]$ (E2)

Clearly, Eq. E2 is describing WVA of small polarization rotation as well as ellipticity effect with simultaneous real and imaginary counterpart. The corresponding expression for the Stokes polarization parameters can beobtained as

$$I=E_{x}{E_{x}}^{*}+E_{y}{E_{y}}^{*}= 2\left( 1+\epsilon_{a}^{2} \right)-2\left( 1-\epsilon_{a}^{2} \right)\left( \cos\alpha\cos\chi\cos2\epsilon_{p}+\sin\alpha\sin\chi\sin2\epsilon_{p} \right)$$

$$Q=E_{x}{E_{x}}^{*}-E_{y}{E_{y}}^{*}=-\left( 1+\epsilon_{a} \right)^{2}\cos2\alpha\cos2\chi-\left( 1-\epsilon_{a} \right)^{2}+2\left( 1-\epsilon_{a}^{2} \right)\left( \cos\alpha\cos\chi\cos2\epsilon_{p}+\sin\alpha\sin\chi\sin2\epsilon_{p} \right)$$

$$U=E_{x}{E_{y}}^{*}+E_{y}{E_{x}}^{*}=\left( 1+\epsilon_{a} \right)^{2}\sin2\alpha\cos2\chi-2\left( 1-\epsilon_{a}^{2} \right)\left( \sin\alpha\cos\chi\cos2\epsilon_{p}-\cos\alpha\sin\chi\sin2\epsilon_{p} \right)$$

$$V=i\left( E_{y}{E_{x}}^{*}-E_{x}{E_{y}}^{*} \right)={-\left( 1+\epsilon_{a} \right)}^{2}\sin2\chi+2\left( 1-\epsilon_{a}^{2} \right)\left( \cos\alpha\sin\chi\cos2\epsilon_{p}+\sin\alpha\cos\chi\sin2\epsilon_{p} \right)$$

(E3)

The expressions for the orientation angle of the polarization vector $\boldsymbol{(}\psi\boldsymbol{)}$ and the circular (elliptical) polarization descriptor 4^th^ Stokes vector element $\left( \frac{V}{I} \right)$can be obtained as

$\psi=\frac{1}{2}{tan}^{-1}\left( \frac{U}{Q} \right)=\frac{1}{2}\tan^{-1} \left( -\alpha\left( 1+\frac{1}{\epsilon_{a}} \right)-\chi\cot\epsilon_{p} \right)\approx-\frac{\alpha}{{2\epsilon}_{a}}-\frac{1}{2}\chi\cot\epsilon_{p}$(E4a)

$\frac{V}{I}\approx\alpha\cot\epsilon_{p}-\frac{\chi}{\epsilon_{a}}$ (E4b)

Simultaneous real and imaginary WVAs are clearly manifested in the$\epsilon_{a}$and $\epsilon_{p}$-dependent terms of the equations respectively. It is also important to note that simultaneous real and imaginary WVA of both Faraday rotation and Faraday ellipticity are manifested in the 1^st^and the 2^nd^ terms of Eq. 4a and 4b respectively. The Stokes vector elements of Eq. E3 were used for the theoretical predictions shown in Figure 2 and Figure 3d of main text on natural WVA of the polarization vector rotation angle$\psi$.

***S3.Numerical Simulation:***

We used the commercially available application software, namely ‘COMSOL Multiphysics’ to implement the Finite element method-based simulation [11]. The simulation was carried out for a given input state of polarization and the transmitted light intensity was analyzed for varying linear and circular polarization states *H, V, P, M, L, R.* This polarization revolved intensities were used to generate 4 Stokes polarization parameters *I, Q, U, V*(as discussed in section S1). A brief overview of the simulation technique is discussed below. One element of the grating period was simulated first and the total field from the full grating was subsequently obtained by applying periodic boundary condition. ACartesian perfectly matched layer (PML) of thickness 200nm was used in the simulation. The PML surrounding our model geometry was placed at a distance of approximately half of the wavelength from the grating structure. The whole geometry including the PML was subsequently divided into domains and sub-domains using free triangular mesh. The mesh element size was chosen to be one fifth of the wavelength while simulating the field outside the grating. For simulating inside the grating, on the other hand, a much finer mesh with a maximum size of 15nm was used to achieve the desirable accuracy of the solution. Finally, we illuminated the whole system and collected the transmittance spectra using COMSOL ports. The inbuilt Port. 1 and Port. 2 of the COMSOL software were employed to generate and analyze the desired polarization states (*H, V, P, M, L, R*) and to subsequently generate the Stokes polarization parameters. The details of the parameters used to simulate the magneto-plasmonic crystal are noted in the main text.

In Figure 3d of the main text, we had shown the exact theoretical prediction (Eq. E3) along with the simulated results for real WVA of Faraday rotation (Eq. E4a). In Figure S2, we summarize the natural WVA of $\frac{V}{I}$Stokes polarization parameter for input TE(y) polarized excitation, from the Fano resonant magneto-plasmonic crystal, results of which were presented in Figure 3 of the main text. Significant enhancement of $\frac{V}{I}$near the spectral window of Fano dip (Fig S2.a) and its comparison with the exact theoretical prediction (Eq. E3) confirms natural WVA of the ellipticity of polarization with varying $\epsilon_{p}\left( \sim\alpha cot \epsilon_{p} \right)$(Eq. E4b).

**
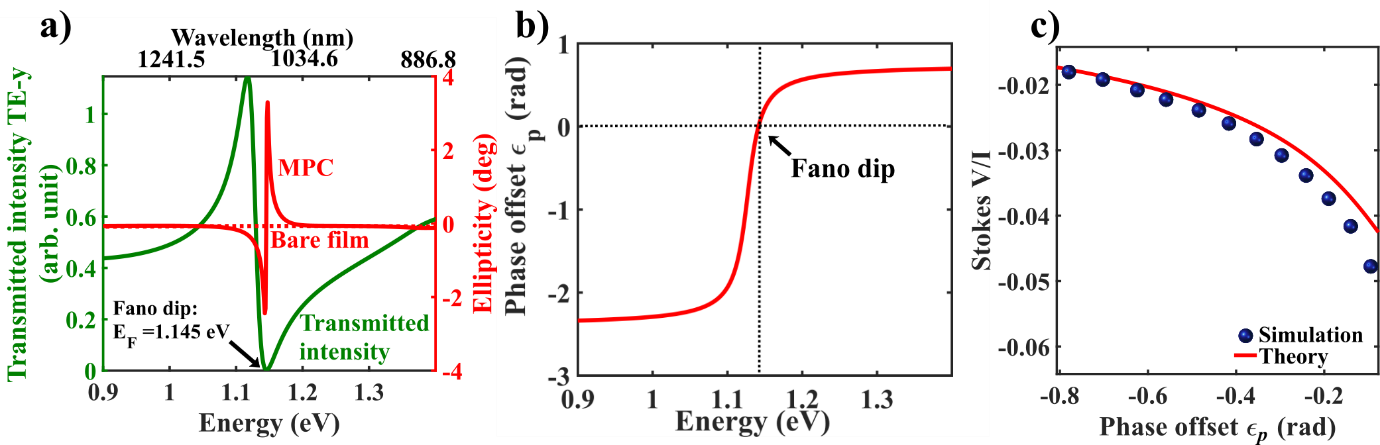
**

**Figure S2: *Natural weak value amplification of*** $\frac{\boldsymbol{V}}{\boldsymbol{I}}$ ***Stokes polarization parameter (ellipticity) in Fano resonant waveguided magneto-plasmonic crystal* (a)** The transmitted intensity (left axis, green line) exhibits prominent Fano spectral asymmetry ($E=\hbar\omega=.9-1.4$ eV, corresponding to $\lambda=1379.4-886.8$nm shown here). The spectralvariation of the $\frac{V}{I}$ Stokes polarization parameter of the magneto-plasmonic crystal (right axis, redsolidline) is depicted along with the bare film Faraday ellipticity(right axis, red-dashed line)**(b)**The spectral variation of phase offset parameter $\epsilon_{p}$.The spectral position of the exact Fano destructive interference is marked. **(c)** The natural weak value amplification of the Stokes polarization parameter$\left( \frac{V}{I} \right)$(blue solid balls) as a function of the phase offset parameter$\epsilon_{p}$ is also plotted with the exact theoretical results (Eq. E3) (redsolid line). The value of rotation and ellipticity of the bare film are$\alpha=0.28$ deg.and $\chi=0.25 deg.$The chosen morphological parameters of the magneto-plasmonic crystal (see main text) resulted in the following parameters of Fano resonance:: $E_{0}=1.127 eV, \gamma=0.0267 eV,q=-1.084, E_{F}=1.141 eV$.

**c)**

**a)**

**c)**

**References**

1. Hosten, O. &Kwiat, P. Observation of the Spin Hall Effect of Light via Weak Measurements. *Science***319**, 787–790 (2008).
2. Pal, Mandira, SudiptaSaha, B. S. Athira, Subhasish Dutta Gupta, and Nirmalya Ghosh. "Experimental probe of weak-value amplification and geometric phase through the complex zeros of the response function." Physical Review A 99, no. 3 (2019): 032123.
3. Gupta, S. D., Ghosh, N. & Banerjee, A. Wave optics: Basic concepts and contemporary trends. (CRC Press, 2015).
4. Ray, S. K. *et al.* Polarization-Tailored Fano Interference in Plasmonic Crystals: A Mueller Matrix Model of Anisotropic Fano Resonance. *ACS Nano***11**, 1641–1648 (2017).
5. Singh, A. K. *et al.*Tunable Fano resonance using weak-value amplification with asymmetric spectral response as a natural pointer. *Phys. Rev. A***97**, 053801 (2018).
6. Ott, C. *et al.* Lorentz Meets Fano in Spectral Line Shapes: A Universal Phase and Its Laser Control. *Science***340**, 716–720 (2013).
7. Ropers, C., et al. "Femtosecond light transmission and subradiant damping in plasmonic crystals." *Physical review letters* 94.11 (2005): 113901.
8. Shcherbakov, M. R., et al. "Ultrafast polarization shaping with Fano plasmonic crystals." *Physical review letters* 108.25 (2012): 253903.
9. Bärnthaler, Andreas, et al. "Probing decoherence through Fano resonances." *Physical review letters* 105.5 (2010): 056801.
10. Gallinet, Benjamin, et al. "Plasmonic radiance: probing structure at the ångström scale with visible light." *Nano letters*13.2 (2013): 497-503.
11. McMahon, J. M. *et al.* Gold nanoparticle dimer plasmonics: finite element method calculations of the electromagnetic enhancement to surface-enhanced Raman spectroscopy. *Anal. Bioanal. Chem.***394**, 1819–1825 (2009).
